# Socializing with Smoker and Social Smoking Behavior among Chinese Male Smokers with Low Nicotine Dependence: The Mediating Roles of Belief of Smoking Rationalization and Smoker Identity

**DOI:** 10.3390/ijerph192214765

**Published:** 2022-11-10

**Authors:** Yuhan Zhang, Jiayu Wang, Keying Lai, Hankun Bian, Haide Chen, Lingfeng Gao

**Affiliations:** 1 College of Education and Human Development, Zhejiang Normal University, Jinhua 321004, China; 2 Key Laboratory of Intelligent Education Technology and Application of Zhejiang Province, Zhejiang Normal University, Jinhua 321004, China

**Keywords:** social smoking behavior, socializing with smokers, belief of smoking rationalization, smoker identity, smokers with low nicotine dependence

## Abstract

Background: Previous studies have shown that socializing with other smokers is an essential trigger for social smoking among smokers with a low nicotine dependence. This study further explored the mediating effects of the belief of smoking rationalization and smoker identity on the relationship between socializing with smokers and social smoking behavior. Methods: A cross-sectional design was conducted. A total of 696 low-nicotine-dependent smokers in China completed questionnaires that assessed socializing with smokers, social smoking behavior, smoker identity, and the belief of smoking rationalization. The mediating roles of the belief of smoking rationalization and smoker identity on the relationship between socializing with smokers and social smoking behavior were assessed by using SPSS 23 and AMOS 23. Results: The belief of smoking rationalization, smoker identity, socializing with smokers, and social smoking behavior were significantly and positively correlated with each other. In addition, this study found an independently mediated role for smoker identity in the relationship with smoker socialization and social smoking behavior, and a sequentially mediated role for smoking rationalization and smoker identity in this relationship. Conclusion: Reducing the belief of smoking rationalization and smoker identity may be conducive to reducing social smoking behavior for low-nicotine-dependent smokers when socializing with other smokers.

## 1. Introduction

Tobacco use is widely recognized as one of the leading causes of preventable diseases and deaths. Smokers with a low nicotine dependence tend to show mild nicotine withdrawal symptoms [1], so, intuitively, they are more likely to choose to quit smoking and quit successfully. However, many smokers with a low nicotine dependence continue to smoke and are not consistently successful in quitting [2,3,4]. One possible reason for this result is that low-nicotine-dependent smokers’ smoking behavior is not only to satisfy nicotine cravings but also to satisfy their psychosocial needs. Social smoking is smoking in social situations, such as parties or hanging out with friends [5]. Existing studies have shown that smokers often smoke in social situations to satisfy the need for better social interaction. This phenomenon is commonly found in smokers with a low nicotine dependence [2,6,7,8]. Reducing social smoking behavior among low-nicotine-dependent smokers may be the key to helping this group quit successfully. Therefore, it is necessary to explore the underlying mechanisms of social smoking behavior generation for smokers with a low nicotine dependence. The present study focused on the effect of socializing with smokers on social smoking behavior and the mediating effects of the belief of smoking rationalization and smoker identity.

### 1.1. Socializing with Smokers and Social Smoking Behavior

Socializing with smokers may have a strong association with social smoking. Socializing with smokers refers to participating in social activities with smokers, such as drinking, gambling, and negotiating with smokers. The Interaction of Person-Affect-Cognition-Execution (I-PACE) model suggests that individuals may perceive stimuli associated with addictive behaviors, which may increase the urge to take addictive behaviors [9]. According to the above theoretical assumptions, the presence of other smokers and their smoking behavior will be the stimuli that induce the smoking behavior of smokers when a smoker participates in an activity with other smokers. Several studies support this view [9,10,11]. For example, previous research has found that ex-smokers are more likely to relapse when they contact smokers [10,11]. In addition, researchers also found that social interaction with other smokers is a crucial factor in motivating individuals to initiate, continue, and restart smoking [12,13,14,15]. Meanwhile, when a group of smokers gathers, smokers usually share cigarettes and persuade each other to smoke, which may cause more social smoking behavior [16]. Based on these theories and empirical evidence, the present study hypothesized that socializing with smokers predicts social smoking behavior.

### 1.2. Socializing with Smokers and Social Smoking Behavior: The Role of Belief of Smoking Rationalization

Although theories and empirical evidence have supported the significant relationships between socializing with smokers and social smoking behavior, there is a limited understanding of its underlying psychosocial mechanisms. Exploring the underlying psychological mechanisms by which socializing with smokers leads to social smoking behavior can help to develop targeted psychological intervention programs to compensate for the inadequacy of pharmacological interventions for social smoking behavior.

Smoking rationalization includes a range of false beliefs about smoking behavior, such as smoking functional belief, risk generalization belief, social acceptability beliefs, safe smoking belief, self-exempting belief, and the belief that quitting is harmful [17]. Some studies have shown that individuals will be more likely to smoke when they have firmer rationalized beliefs about smoking [18,19]. The theory of normative social behavior (TNSB) [20] suggests that when people perceive the widespread presence of smokers, they will consider smoking as a behavioral norm and see it as a reasonable, socially acceptable, and harmless behavior. Some evidence supports this view. For example, Huang and his colleagues found that smoking in China is a common phenomenon, and therefore people think that smoking is commonly accepted [17,19]. Meanwhile, one instance of research in Indonesia has also shown that an individual will think that smoking is acceptable to society when many smokers are around them [21]. According to empirical studies and TNSB, when a smoker is more frequently involved in social activity with other smokers, the smoker is also more exposed to smoking and is more likely to hold the belief of smoking rationalization. Furthermore, this belief may be the cognitive factor that mediates environmental factors and smokers’ smoking behavior. Hence, the present study hypothesized that the relationship between socializing with smokers and social smoking behavior might be mediated by the belief of smoking rationalization.

### 1.3. Socializing with Smoker and Social Smoking Behavior: The Role of Smoker Identity

Smoker identity is another possible mediator between socializing with smokers and social smoking behavior. Smoker identity is defined as the degree that smokers regard smoking behavior as a crucial part of their self-identification [22]. Empirical research found that the more smokers identify themselves as smokers, the more cigarettes they smoke [23,24,25,26,27,28]. According to the recent theory of the Social Identity Model of Cessation Maintenance (SIMCM) [29], when smokers frequently contact other smokers, they are more likely to identify themselves as ‘smokers’ and to smoke more heavily. Some studies have shown that the more addicts that exist in a recovering addict’s social network, the more challenging it is for the recovering addict to change the addict identity and the higher the probability of relapse [30,31,32]. According to these theories and empirical evidence, smokers who regularly attend social events with other smokers are more likely to identify themselves as smokers and smoke more cigarettes. Hence, the present study hypothesized that the relationship between socializing with smokers and social smoking behavior is mediated by the smoker identity.

### 1.4. Socializing with Smokers and Social Smoking Behavior: The Roles of Belief of Smoking Rationalization and Smoker Identity

Based on those mentioned theoretical and empirical studies, we expected the relationship between socializing with smokers and social smoking behavior to be sequentially mediated by the belief of smoking rationalization and smoker identity. The social identity theory (SIT) suggests that an individual decides whether or not to adopt group-approved behaviors by evaluating their beliefs and the importance of their social group to themself [33]. Similarly, individuals are more likely to identify with a group when they believe fitting in is harmless or beneficial. Based on SIT and SIMCM, smokers’ belief of smoking rationalization may influence their identity as ‘smokers’. Some indirect evidence supports this view. For example, previous research has found that smoker identity is influenced by society’s evaluation of smokers. If smoking is considered acceptable, smokers will strengthen their identity as smokers [34,35,36,37,38]. However, smokers tend to refuse to identify themselves as smokers if they perceive negative social attitudes toward smoking [39]. According to the above theoretical and empirical evidence, the present study hypothesized that smokers’ belief of smoking rationalization might be influenced by socializing with smokers, which may affect smokers’ self-identity and, in turn, affect social smoking behavior.

### 1.5. The Current Study

This study examined the association between socializing with smokers and social smoking behavior among low-nicotine-dependent male smokers in China and the independent and sequentially mediating effect of the belief of smoking rationalization and smoker identity on this relationship. We focused on male rather than female smokers for two reasons. First, only 2.1% of Chinese women are smokers (Chinese Center for Disease Control and Prevention, 2018). Second, in China, smoking has traditionally been considered a symbol of masculinity and is exclusively reserved for men. Female smokers tend to feel more ashamed and experience more significant stigma about smoking [40,41]. These situations make recruiting a large sample of female regular smokers difficult.

Based on these theories and empirical evidence, the present study proposed the following specific hypotheses:

**Hypothesis** **1** **(H1).** *Social smoking behavior among Chinese male smokers with a low nicotine dependence is positively predicted by socializing with smokers*.

**Hypothesis** **2** **(H2).** *The relationship between socializing with smokers and social smoking behavior is mediated by the belief of smoking rationalization*.

**Hypothesis** **3** **(H3).** *The relationship between socializing with smokers and social smoking behavior is mediated by smoker identity*.

**Hypothesis** **4** **(H4).** *The relationship between socializing with smokers and social smoking behavior is sequentially mediated by the belief of smoking rationalization and smoker identity*.

**Figure 1:**
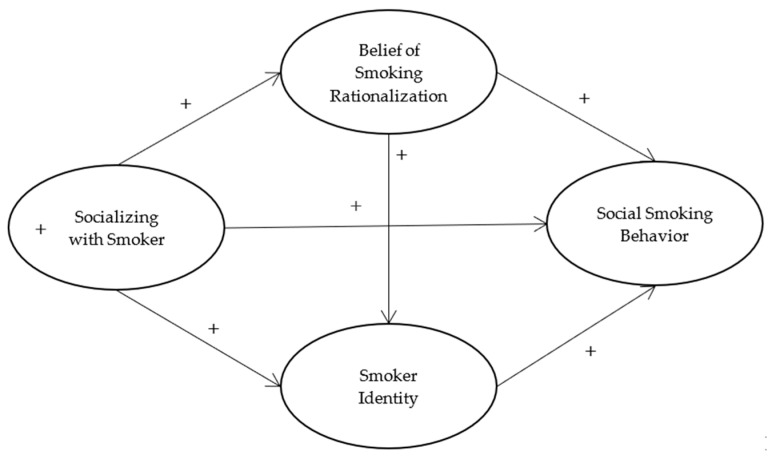
Conceptual model.

The hypothesized model is shown in Figure 1.

## 2. Materials and Methods

### 2.1. Procedures and Participants

The institutional ethical review board of the researchers approved the study before participants answered the questionnaire, which consisted of their demographic information. Recruitment advertisements were posted on the community’s social networking sites and bulletin boards, including information about the project, survey requirements, and benefits of participation. After contacting, the participants were first required to answer three questions before they were allowed to participate in the assessment (i.e., “How old did you start smoking?” “Have you smoked at least 100 cigarettes in your life?” “How many cigarettes have you smoked per day in the past month?”). According to these, this study recruited 1234 participants who had smoked more than two years, had smoked more than 100 cigarettes in total, and had smoked more than 3 cigarettes per day. The survey was completed anonymously through an online survey management system. The valid sample consisted of 1016 participants after deleting the 218 participants who had missed answers, answered too fast, or regularly answered in the responses. There were 924 men among all participants. In this study, 696 (75.3%) male smokers with low nicotine dependence who scored less than or equal to 5 on the Fagerstrom Test for Nicotine dependence (FTND) in the sample were selected as the study subjects [42]. The FTND was completed simultaneously as the subject participated in the survey. Participants smoked an average of 9.23 (SD = 6.71) cigarettes per day. The age range was from 18 to 61 years (M = 30.43, SD = 10.14), and the mean score of the socioeconomic status t was 10.41 (SD = 3.26). In terms of marital status, 406 (58.3%) of participants were unmarried, whereas 290 (41.7%) were married.

### 2.2. Measures

#### 2.2.1. Socializing with Smokers

This study summarized the common social behavior closely related to smoking based on the results of social surveys and some empirical studies [43,44], and compiled a socializing with smokers scale. This scale consisted of 5 items (e.g., “Drinking with a friend (or leader) who smokes.”, “Gambling with a friend (or leader) who smokes.”, “Singing at KTV with a friend (or leader) who smokes.”, “Discuss the cooperation with a friend (or leader) who smokes.”). Responses were recorded on a 4-point Likert scale that ranged from 1 (never) to 4 (always). The higher average scores indicate a higher frequency of social events with other smokers. The result of CFA showed that χ^2^/df = 4.25, AGFI = 0.96, TLI = 0.97, RMSEA = 0.07(95%CI = 0.02, 0.12). The Cronbach’s α of the scale was 0.81 in the present study.

#### 2.2.2. Social Smoking Behavior

This study compiled a social smoking behavior scale based on existing studies [8,45]. The scale consisted of 6 items (e.g., “I smoke when drinking with friends.”, “I smoke when I am talking with friends”, “I smoke when I am gambling with friends”, and “I smoke if I have a new friend and pass cigarettes to each other”). Responses were recorded on a 4-point Likert scale that ranged from 1 (never) to 4 (always). The higher average scores indicate a higher frequency of social smoking. The result of CFA showed that χ^2^/df = 1.39, AGFI = 0.99, TLI = 0.99, RMSEA = 0.02 (95%CI = 0.00, 0.07). The Cronbach’s α of the scale was 0.86 in the present study.

#### 2.2.3. Belief of Smoking Rationalization

The belief of smoking rationalization was measured using the smoking rationalization scale for smokers in China [19]. The scale consisted of 6 dimensions (smoking functional belief, risk generalization belief, social acceptability beliefs, safe smoking belief, self-exempting belief, and the belief that quitting is harmful) and 26 items (e.g., “Smoking is normal for men.”, “Smoking can reduce interpersonal distance and make social interaction easier.”, “Many famous people smoke, so it is normal to smoke.”, “There are so many smokers in society, so it is hard for you to be different.”, “If smoking was so bad for health, the government would have banned tobacco sales.”) in total. Responses were recorded on a 5-point Likert scale that ranged from 1 (strongly disagree) to 5 (strongly agree). The higher average scores indicate that smokers believe that smoking is acceptable to themselves and society. The result of CFA showed that χ^2^/df = 2.97, AGFI = 0.97, TLI = 0.98, RMSEA = 0.05 (95%CI = 0.02, 0.09). The Cronbach’s α of the scale was 0.81 in the present study.

#### 2.2.4. Smoker Identity

Smoker identity was measured using an adaptation of the measure developed by Moan and colleagues [46]. The scale consisted of 4 items (e.g., “I am a good example of a person who smokes.”, “I consider myself a smoker.”, and “If I do not smoke, I feel like I have lost something.”). Responses were recorded on a 7-point Likert scale that ranged from 1 (strongly disagree) to 7 (strongly agree). The higher average scores indicate a higher level of smoker identity. The result of CFA showed that χ^2^/df = 1.30, AGFI = 0.99, TLI = 0.99, RMSEA = 0.02 (95%CI = 0.00, 0.07). The Cronbach’s α of the scale was 0.79 in the present study.

#### 2.2.5. The Fagerstrom Test for Nicotine Dependence

Nicotine dependence was measured using the Fagerstrom Test for Nicotine Dependence [42]. The scale consisted of 6 items (e.g., “In the last month, how many cigarettes did you smoke on average per day?”). Composite scores, ranging from 0 to 10, can be computed by summing the individual item scores. Higher scores are indicative of greater nicotine dependence among smokers. The result of CFA showed that χ^2^/df = 2.17, AGFI = 0.98, TLI = 0.85, RMSEA = 0.04 (95%CI = 0.00, 0.09). The Cronbach’s α of the scale was 0.65 in the present study.

#### 2.2.6. Socioeconomic Status

The subjects’ socioeconomic status was measured using the objective socioeconomic status scale [47]. The scale consisted of 6 items (e.g., “How much CNY 10,000 a year is your disposable income?”, and “What is your occupation?”). Composite scores, ranging from 3 to 21, can be computed by summing the individual item scores. Higher scores are indicative of higher socioeconomic status (SES) among smokers.

### 2.3. Data Analysis

All analyses were conducted using SPSS 23.0 and AMOS 23.0 (SPSS: Armonk, NY, USA). We divided the data analysis into two parts. First, we conducted the descriptive statistics and correlations between the variables. Second, we tested the hypothesized multiple mediation model by using structural equation modeling (SEM). The data used in the analysis are the means of the participants’ scores on the scales. Participants’ scores on the scales were treated as continuous data in the analysis.

## 3. Results

### 3.1. Common Method Bias

Due to the limitation of the self-reported data collection method, the Harman common method variance (CMV) was tested first. The results showed that there were ten factors with characteristic roots more significant than 1, and the first factor explained 19.55% of the critical standards, which indicated that the common method has no significant bias on the current results of this study.

### 3.2. Descriptive Statistics

The descriptive statistics and correlations among the main variables are shown in Table 1. The results of partial correlation analyses (i.e., controlling for the age and socioeconomic status of the subjects) showed a significant positive correlation among all variables, which is in line with our hypotheses.

### 3.3. The Mediating Effects of Belief of Smoking Rationalization and Smoker Identity

We conducted a mediation analysis with AMOS. After controlling for the smoker’s age and socioeconomic status, we selected 2000 bootstrap samples from the original data (N = 696) by repeating the random sampling method to establish the multiple mediation effect model (χ^2^/df = 2.26, CFI = 0.97, TLI = 0.95, RMSEA = 0.04), and the model fit well. As displayed in Table 2, the total effect of socializing with smokers on social smoking behavior was positive (β = 0.54, 95%CI [0.45, 0.61]), and the total indirect effect was significant (β = 0.12, 95%CI [0.08, 0.16]). It explained 22.22% of the total effect. Specifically, smoker identity independently mediated the relationship between socializing with smokers on social smoking behavior (β = 0.10, 95%CI [0.06, 0.14]). It explained 18.54% of the total effect. In addition, the belief of smoking rationalization and smoker identity also sequentially mediated this relationship (β = 0.02, 95%CI [0.00, 0.03]). It explained 3.70% of the total effect.

**Figure 2:**
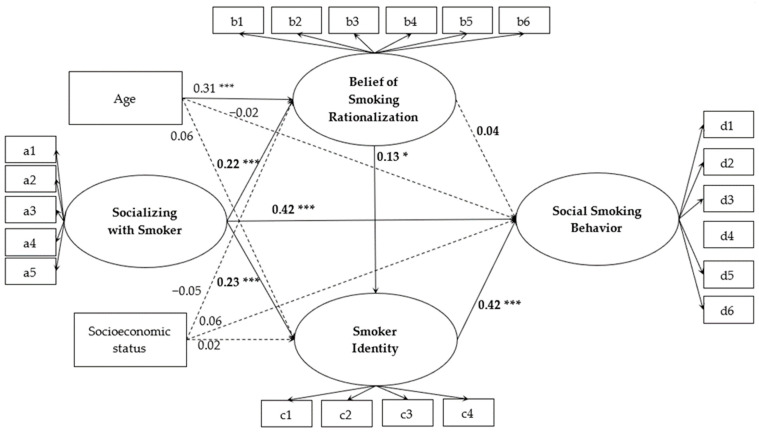
The finalized structural model (factor loadings are standardized, N = 696). Note: * *p* < 0.05, *** *p* < 0.001.

As shown in Figure 2, socializing with smokers positively predicted smokers’ belief of smoking rationalization (β = 0.23, 95%CI [0.15, 0.31]) and smoker identity (β = 0.23, 95%CI [0.14, 0.31]). In turn, smokers’ smoker identity positively predicted social smoking behavior (β = 0.42, 95%CI [0.33, 0.50]). In addition, smokers’ belief of smoking rationalization was positively linked to smoker identity (β = 0.13, 95%CI [0.03, 0.23]).

## 4. Discussion

The present study proposed that the belief of smoking rationalization and smoker identity would sequentially mediate the relation between socializing with smokers and social smoking behavior. As hypothesized, socializing with smokers predicted social smoking behavior, which was explained by the sequentially indirect effect of the belief of smoking rationalization and smoker identity. Specifically, an increased socialization with smokers was correlated with an increased belief of smoking rationalization, which was related to an increased smoker identity and, in turn, more social smoking behavior. Smoker identity independently accounted for part of the relationship between socializing with smokers and social smoking behavior. These results underscore the importance of the belief of smoking rationalization and smoker identity as potential factors in explaining the relationship between socializing with smokers and social smoking behavior among Chinese male smokers with a low nicotine dependence.

### 4.1. Relationship between Socializing with Smokers and Social Smoking Behavior

Consistent with previous research [10], the present study found that social smoking behavior was significantly and positively correlated with socializing with smokers, supporting H1. Smokers who attend social events more frequently with other smokers are more likely to engage in social smoking behavior. These results might be explained by the I-PACE model. The I-PACE model assumes that addiction-related cues in the addict’s surroundings can induce a range of physical and psychological responses, including craving, that lead to addictive behavior [9]. According to this assumption, smoking-related cues may induce smoking cravings and smoking behavior in smokers. When smokers socialize with other smokers, even in the absence of cigarettes and actual smoking behavior, the other smokers may become a smoking-related cue, inducing cravings and leading to social smoking [10]. In addition, it is known that persuading others to use tobacco often occurs in the social interactions between smokers. This behavior is also a risk for social smoking.

### 4.2. The Mediating Role of Smoker Identity

The present study found that the relationship between socializing with smokers and social smoking behavior was partially mediated by smoker identity, supporting H3. This result is similar to previous findings [28,32,34]. According to this result, the reason why smokers smoke when socializing with other smokers may not only be caused by the physiological craving for nicotine that smokers generate but may also be caused by the activated smoker identity, which generates the need to belong to a group of smokers. These results can be explained as follows. Firstly, existing theories of addict identity emphasize that addict identity is a crucial personality characteristic causing smoking behavior, and situational factors can influence addictive behavior by influencing addict identity [29,48]. Smokers are more likely to be exposed to smoking-related cues when they are involved in social situations where other smokers are present [49]. In such situations, smokers may activate their own smoker identity, thus leading to social smoking behavior. Secondly, many studies have shown that, in order to strengthen their ties to the group and to satisfy their need to belong, individuals tend to behave in a way that conforms to the norms of the group to which they belong [50,51]. When a group of smokers is together, they are more likely to smoke to strengthen their connection to the group and satisfy their need to belong.

### 4.3. Sequential Mediating Effect of Belief of Smoking Rationalization and Smoker Identity

This study also found that the relationship between socializing with smokers and social smoking among Chinese low-nicotine-dependent male smokers could be explained by the sequential mediating effect of the belief of smoking rationalization and smoker identity. According to the assumptions of SIT and SIMCM [29,33], this finding may be explained as follows. First, smokers are more likely to generate firmer beliefs about the social acceptability of smoking when socializing with other smokers [17,19]. Next, this belief may lead smokers to identify more with themselves as smokers to better fit into the social situation [36]. Finally, a high smoker identity activated in the social environment may lead to social smoking behavior [23]. In other words, regular socialization with other smokers may lead the smoker to believe that smoking is reasonable, thus enhancing the smoker’s identity. Further, to maintain their self-identity and satisfy their need for a sense of belonging, the smoker is more likely to smoke to socialize.

In addition, the results of this study found that the effect of socializing with smokers and social smoking behavior was mediated primarily by identity (20% of the total effect), that socializing with smokers was a significant predictor of smoking rationalization beliefs, and, in turn, that smoking rationalization was a significant predictor of smoker identity, but that the independent mediators of smoking rationalization were not significant and that the sequential mediating effect of smoking rationalization and smoker identity, although significant, accounted for a relatively small proportion of the total effect. This study considers the following as possible causes for this phenomenon. Firstly, frequent socializing with smokers may lead to individuals repeatedly fitting in with the smoker group, thus developing a strong sense of belonging to the smoker group and consequently leading individuals to smoke. Second, the effect of smoking rationalization on smoker identity and social smoking behavior may be influenced by the group of subjects. According to SIMCM theory [33], the emergence of an addict’s identity is a staged process; that is, the identity of an addict is gradually formed and consolidated. This study hypothesized that smoking rationalization might have a more significant impact on the individual smoker identity and social smoking behavior when the individual has just started smoking, which is the early stage of the emergence of smoker identity, whereas, after the individual has become a daily smoker, smoking rationalization only has a consolidating effect on the smoker identity After the smoker has become a daily smoker, smoking rationalization only consolidates but no longer promotes smoker identity. The subjects in this study were daily smokers, not occasional or social smokers. Further research is needed in future studies to examine the role of smoking rationalization in different types of smoker groups.

## 5. Contributions and Limitations

The current study may offer several implications for smoking research. Firstly, based on the theoretical view that social cognition influences self-concept formation, this study reveals the independently mediated role of smoker identity in relation to socializing with smokers and social smoking behavior, and the sequentially mediated role of smoking rationalization and smoker identity in this relationship. These findings shed light on the underlying mechanism between the social context surrounding smokers and social smoking behavior, highlight the critical role of social cognition and self-concept in explaining social smoking behavior, and complement I-PACE and SIMCM. Secondly, the research focused on low-nicotine-dependent male smokers, which contributed to a targeted review of the psychosocial mechanisms that explain this group’s difficulty in quitting smoking and provided theoretical guidance for developing effective intervention programs. Finally, it provided enlightenment to the work of tobacco control. On the one hand, instead of nicotine replacement therapy (NRT), intervention, including psychosocial factors, should lead low nicotine-dependence smokers to participate in cessation groups and encourage them to form a new identity as a quitter through experience sharing within the groups. On the other hand, it is also necessary to change smokers’ irrational perception of the social value of cigarettes, coupled with social skills training for quitting, and to use other more positive ways to build strong social bonds with others.

Despite the contributions mentioned above, some significant limitations remain to be considered. First, the sample used in this study consisted of only male smokers, so the effects may not be universal. Future studies should balance the proportion of males and females and increase the sample size from different cultures. Secondly, a cross-sectional design was used to explore the underlying mechanism of socializing with smokers in social smoking behavior, which cannot answer the causal relationship between variables strictly. Since a cross-sectional design cannot identify the order of events/behaviors, it will be necessary to test the causal relationships between socializing with smokers, smoking rationalization, smoker identity, and social smoking behavior by using a longitudinal design or laboratory experiments in future studies. Thirdly, the measurement of socializing with smokers and social smoking behavior was limited because the questionnaire developed for this study was not adequately validated. Finally, a self-reporting measure used in our study could make the results become affected by social desirability. Although the common method deviation of this study does not reach a significant level, future studies should try to collect data from multiple channels to confirm the findings.

## 6. Conclusions

This study found that the relationship between engagement with smokers and social smoking behavior could be mediated by smoker identity in an independently mediated manner and by smoking rationalization beliefs and smoker identity in a sequentially mediated manner. Our study suggests that reducing smoking rationalization beliefs and smoker identity may contribute to a reduction in social smoking among smokers with a low nicotine dependence.

## Figures and Tables

**Table 1:** Mean scores, standard deviations, and Pearson’s correlations of the study variables.

Variables	M ± SD	1	2	3	4
1. Socializing with Smokers	2.23 ± 0.59	-			
2. Social Smoking Behavior	2.73 ± 0.70	0.46 ***	-		
3. Belief of Smoking Rationalization	2.80 ± 0.58	0.20 ***	0.21 ***	-	
4. Smoker Identity	4.56 ± 1.21	0.19 ***	0.43 ***	0.17 ***	-

Note. *** *p* < 0.001.

**Table 2:** Standardized estimates and 95% CIs for direct and indirect effects.

Pathways	Estimate	95%CI	
		Lower	Upper
Direct effects			
Socializing with Smoker → Social Smoking Behavior	0.42	0.33	0.50
Total indirect effect			
Socializing with Smoker → Social Smoking Behavior	0.12	0.08	0.16
Specific indirect effect			
Socializing with Smoker → Belief of Smoking Rationalization → Social Smoking Behavior	0.01	−0.01	0.03
Socializing with Smoker → Smoker Identity → Social Smoking Behavior	0.10	0.06	0.14
Socializing with Smoker → Belief of Smoking Rationalization → Smoker Identity → Social Smoking Behavior	0.02	0.00	0.03

## Data Availability

The datasets analyzed during the current study are available from the corresponding author on reasonable request.
